# Feasibility of Intraoperative SS-OCT for Assessing Capsular-Bag Dynamics During Cataract Surgery: Pilot Randomized Intra-Individual Comparison of Adaptive Versus Gravity-Based Fluidics

**DOI:** 10.3390/diagnostics16142282

**Published:** 2026-07-21

**Authors:** Stefan Georgiev, Stefan Palkovits, Andreea Dana-Fisus, Manuel Ruiss, Caroline Pilwachs, Christoph Leisser, Oliver Findl

**Affiliations:** Vienna Institute for Research in Ocular Surgery (VIROS), A Karl Landsteiner Institute, Hanusch Hospital, 1140 Vienna, Austria

**Keywords:** adaptive fluidics, biometry, optical coherence tomography, cataract surgery

## Abstract

**Background/Objectives**: This exploratory, randomized, patient-masked, intra-individual pilot study evaluated the feasibility of intraoperative swept-source optical coherence tomography (SS-OCT) for quantifying capsular-bag dynamics during cataract surgery and explored potential differences between an adaptive fluidics system (AFS) and a gravity-based fluidics system (GFS). **Methods**: Twenty-seven myopic cataract patients (54 eyes) underwent bilateral phacoemulsification, with one eye randomly assigned to AFS and the fellow eye to GFS using the same phacoemulsification platform (Stellaris/Stellaris Elite, Bausch & Lomb, Rochester, NY, USA). Intraoperative SS-OCT scans were acquired at six predefined surgical stages (M1–M6) to measure anterior and posterior capsule distances relative to the corneal endothelium. Intraoperative discomfort was assessed using a visual analog scale. **Results**: Of 648 attempted scans, 392 (60.5%) were analyzable, confirming partial feasibility of the prototype system. At the onset of phacoemulsification with irrigation on (M2), the predefined primary endpoint, anterior-capsule distance was greater with GFS than with AFS (4.23 ± 0.58 mm vs. 3.72 ± 0.73 mm), corresponding to greater mean deepening from baseline under GFS (1.19 mm) than under AFS (0.63 mm). However, this difference was not statistically significant after adjustment for multiple comparisons. No significant inter-system differences were found at other timepoints, and overall discomfort scores were similar (*p* = 0.70). **Conclusions**: Intraoperative SS-OCT enabled real-time visualization and partial quantitative assessment of capsular-bag dynamics during cataract surgery. The observed difference at the initiation of phacoemulsification may suggest reduced anterior-capsule deepening with adaptive fluidics, but this comparison was not statistically significant after multiplicity adjustment. The 39.5% measurement failure rate underscores the need for technical refinement before routine clinical implementation.

## 1. Introduction

Cataract surgery, as one of the most common procedures, invariably entails the surgical removal of the opacified lens and its replacement with an artificial intraocular lens (IOL) implant. Along with continuous improvements, phacoemulsification as the prevailing method has reached a considerable level of maturity leading to predictable postoperative visual outcomes and low complication rates [1]. For decades, intraoperative gravity fluidics systems (GFSs) have been the standard choice to induce continuous intraoperative irrigation pressure. Hereby, the difference between a specified balanced salt solution (BSS) bottle height relative to the patient’s eye creates an intraoperative irrigation pressure gradient to maintain a pressurized anterior chamber during the procedure. Nevertheless, throughout surgery, the fluid aspiration rate constantly changes, whereas the static BSS bottle with fixed irrigation pressure is usually positioned as high as necessary to account for the maximum amount of vacuum and prevent anterior chamber surge. As such, unnecessary high intraocular pressure (IOP) can occur during the surgical procedure [2]. Moreover, to maintain a stable fluid inflow/outflow balance, and thus, anterior chamber depth (ACD) stability, the bottle height needs to be adjusted. However, due to frequent aspiration rate changes, this adjustment may fail to consistently occur in a timely manner. The delayed modulation can not only disrupt surgical workflow but also cause IOP variability associated with ACD fluctuations causing intraoperative adverse events [3,4]. These include occlusion break surges that may increase the risk of posterior capsule ruptures, decreased retinal blood flow, patient discomfort, and postoperative complications due to intraocular tissue injuries [5].

At the beginning of the last decade, active and adaptive fluidics systems were introduced to address this issue with the aim of maintaining physiological IOP levels throughout surgery [6,7,8]. In active fluidics, a BSS bag as the irrigation source is placed into a phacoemulsification machine equipped with pressure sensors to continuously monitor the IOP. When the IOP drops below the predetermined threshold, the BSS bag is compressed to elevate the irrigation pressure. Conversely, when the IOP exceeds the nominal preset value, the BSS bag is decompressed to lower the IOP. In adaptive fluidics systems (AFS) on the other hand, IOP is proactively regulated using air pressure in the bottle in accordance with the commanded vacuum level pressure. As such, both systems have a premise to maintain a stable ACD despite fluctuating aspiration rates.

However, conclusive large-scale clinical trials remain to be conducted, even if its benefits have been previously discussed for patients with coexisting morbidities/conditions (e.g., keratopathy, diabetes) [9,10,11], short axial length (AL) with shallow ACD [12], or myopic eyes less tolerant to trauma due to a floppier iris and/or capsular bag with looser zonules, and a higher propensity for lens–iris diaphragm retropulsion [13,14,15]. The latter—characterized by posterior displacement of the iris–lens diaphragm, excessive anterior chamber deepening, and patient discomfort—is particularly common in myopic eyes with long axial length and lax zonules. Because such eyes are prone to exaggerated intraoperative pressure fluctuations, they provide an ideal model for studying capsular-bag dynamics under different fluidics conditions.

More recently, the use of swept-source optical coherence tomography (SS-OCT) to acquire precise full eye-length biometric parameters has become increasingly used in cataract surgery [16,17]. As a non-invasive cross-sectional imaging technique, intraoperative OCT has been further successfully employed for visualizing anterior chamber structures, the capsular bag, IOL positioning, residual lens fragments, and measuring AL and other intraocular distances [18,19,20,21]. Nonetheless, although SS-OCT engines have advantages in terms of signal quality and imaging depth, they are yet to be implemented as intraoperative commercial systems [22].

On that ground, using an intraoperative SS-OCT prototype, the following pilot study was designed to comparatively evaluate the impact of GFS (Stellaris, Bausch & Lomb, Rochester, NY, USA) versus its successor AFS module (Stellaris Elite, Bausch & Lomb, Rochester, NY, USA) on intraoperative capsular bag fluctuation in myopic cataract patients. The aim was to assess the feasibility of performing axial capsular bag measurements at various timepoints during cataract surgery, especially during phacoemulsification and irrigation/aspiration, in order to evaluate whether intra-individual differences in capsular-bag dynamics between GFS and AFS can be delineated.

## 2. Materials and Methods

### 2.1. Study Design

This pilot randomized, patient-masked, single-center, intra-individual (paired-eye), controlled study (NCT identifier: NCT03751254, registered 23 November 2018) enrolled myopic patients (AL > 25 mm) affected by bilateral visually significant cataracts scheduled for surgery at the department of ophthalmology of the Hanusch hospital in Vienna. The study adhered to the principles of the Declaration of Helsinki and received approval from the local ethics committee (EK 18-135-0818), with signed written informed consent having been obtained from all participants prior to surgery.

**Figure 1:**
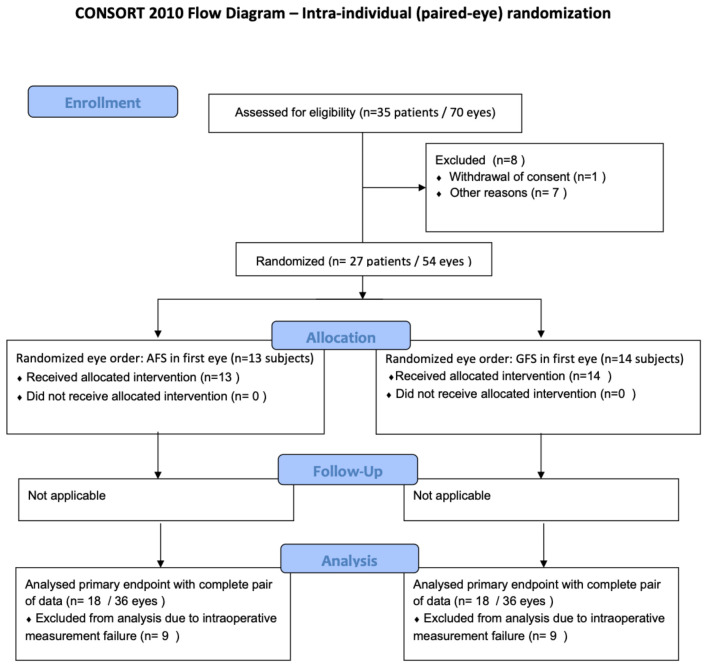
Flowchart of the randomized intra-individual (paired-eye) trial comparing adaptive fluidics (AFS) and gravity-based fluidics (GFS). Thirty-five patients (70 eyes) were screened; eight were excluded. Twenty-seven patients (54 eyes) were randomized, with one eye receiving AFS and the fellow eye GFS. For the primary intraoperative endpoint (M2 = phacotip insertion with activated irrigation), complete paired data were available for 18 pairs (36 eyes).

Exclusion criteria were prior ocular surgery or trauma, corneal opacities, or abnormalities that could compromise measurements and/or impair fixation stability for biometric measurements (e.g., strabismus, amblyopia, iris pathologies). In addition, successful acquisition of preoperative SS-OCT-based optical biometry was required for study inclusion; therefore, eyes with lens opacities severe enough to preclude reliable optical biometry (mature cataracts) were not eligible. A 1:1 block randomization (http://www.randomizer.org, accessed on 30 November 2018) was used, whereby the first eye to be operated was randomly allocated either into the GFS or the AFS arm (Figure 1). The surgery of the contralateral eye was performed 1 to 2 weeks after operation of the first eye with the other fluidics system, thereby enabling a paired within-subject comparison of GFS versus AFS.

### 2.2. Preoperative Examination

Baseline measurements 1 week prior to surgery included a full ophthalmic assessment via slit lamp examination and SS-OCT-based optical biometry scans at 1055 nm center wavelength (IOLMaster 700, Carl Zeiss Meditec AG, Jena, Germany) to derive AL, ACD, lens thickness (LT) and keratometry (K) readings.

### 2.3. Surgical Procedure

Three experienced surgeons performed standard phacoemulsification under topical anesthesia. The procedure included a clear corneal incision using a single-beveled steel blade, ophthalmic viscoelastic device (OVD) injection, hydrodissection, phacoemulsification, co-axial irrigation/aspiration of cortical remnants, posterior capsule polishing, OVD re-injection, IOL implantation in the capsular bag, and OVD aspiration, followed by stromal wound hydration. The same open-blade lid speculum by Lieberman (Geuder AG, Heidelberg, Germany) was utilized, and a monofocal aspheric CT Asphina 409 IOL (Carl Zeiss Meditec AG, Germany) was implanted in all cases.

For this study, patients were masked to which fluidics system was used in which eye, and GFS/AFS were randomized to both eyes according to the randomization pattern as shown in Figure 1. In the AFS arm, eyes were operated with the Stellaris Elite (Bausch & Lomb, Rochester, NY, USA) phacoemulsification system with target IOP set at 20 mmHg. It consists of a BSS glass bottle which hangs much lower than in conventional GFS. Air from a pneumatic compressor flows into the bottle through the sterile tubing connected to it. Infusion pressure is adjusted according to the real-time surgeon-commanded vacuum level. This is intended to maintain more consistent pressure conditions and reduce anterior-chamber fluctuations. In the GFS arm, the same machine was used, albeit with the conventional irrigation infusion bottle height set at 100 cm height (equivalent to IOP target of about 70 mmHg).

In both groups, I/A vacuum level and aspiration flow levels were set at 400 mmHg and 40 cc/min, respectively. Throughout phacoemulsification, study-related measurements consisted of several intraoperative SS-OCT biometry scans (as explained below). Additionally, patients’ subjective discomfort was assessed at different timepoints during phacoemulsification and irrigation/aspiration using a visual analog scale (VAS) score ranging from lowest discomfort (0) to highest discomfort (10). Routine postoperative care remained unaltered, with patients having received bromfenac (Yellox, Bausch & Lomb, Bridgewater, NJ, USA) twice daily for 1 month.

### 2.4. Intraoperative SS-OCT Measurements

**Figure 2:**
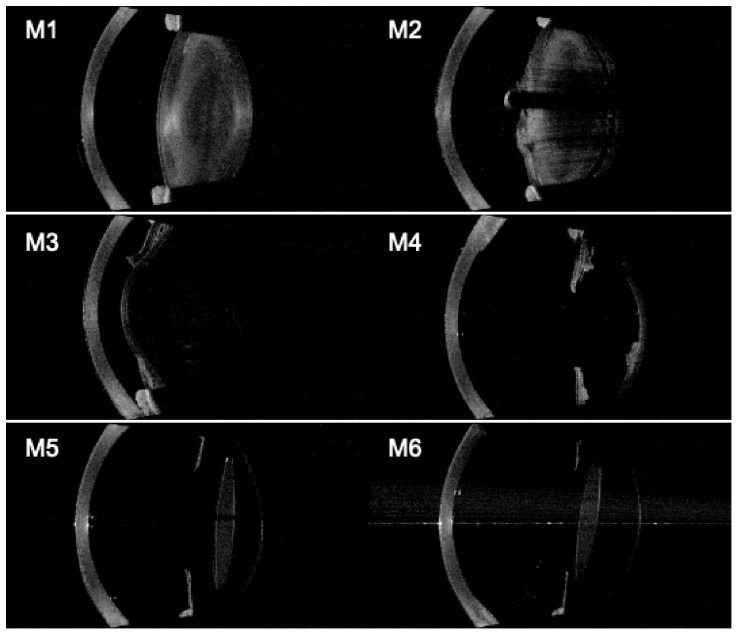
Intraoperative axial iOCT measurements at different timepoints throughout phacoemulsification. Upper row: left phakic baseline measurement (**M1**), right post-rhexis and hydrodissection with phacotip inserted and irrigation on (**M2**). Middle row: left aphakic measurement before (**M3**) and right after I/A tip insertion (**M4**). Bottom row: left pseudophakic measurement before (**M5**) and right after I/A tip insertion (**M6**).

Intraoperative SS-OCT (iOCT) measurements were performed with a prototype previously evaluated by our group for AL measurements [19]. The IOLMaster 700 biometer connected to an OPMI Lumera 700 microscope (Carl Zeiss Meditec AG) was used to acquire intraoperative biometric measurements at 6 timepoints in the phakic, aphakic, and pseudophakic state (see Figure 2). Specifically, after draping and lid speculum placement, an initial baseline iOCT measurement was acquired (M1), followed by a clear corneal incision and OVD injection. After capsulorhexis and hydrodissection, a second scan was acquired just after entering with the phaco-probe with irrigation on (M2). After phacoemulsification, two measurements were acquired in the aphakic state. M3 was obtained before I/A tip insertion, and M4 after insertion with irrigation on. The final two scans were performed after IOL implantation before (M5) and immediately after (M6) I/A tip insertion with irrigation on. In addition, patients were asked to describe their subjective discomfort using a VAS score at measurement timepoints M2, M4, and M6, as well as their overall pain perception immediately after surgery.

### 2.5. Data Processing

Pseudonymized preoperative IOLMaster 700 and intraoperative SS-OCT scans were imported into ImageJ software version 1.54j (National Institutes of Health, Bethesda, MD, USA) to measure intraocular distances in pixels [23]. To do so, the straight-line function in ImageJ was applied to the referential preoperative scan of the conventional IOLMaster 700. The pixel-wise ACD distance was converted into mm based on the nominal value from the investigational software of the commercial biometer. Standardized distances were then applied to the intraoperative SS-OCT scans using the ImageJ known distance function. For an initial validation, the strength of the correlation between preoperative ACD measurements and the respective intraoperative baseline ACD values was assessed. From thereon, similar to a previous trial by our group [24], the axial distances of all intraoperative measurements were determined between the corneal endothelium and the anterior and posterior capsules. Since the anterior lens capsule could not be directly measured after capsulorhexis, a perpendicular line from the corneal center in the x–y axis to the center line between both rhexis edges was used for analysis. For the posterior capsule, the distance between the corneal center in the x-y axis and the center of the posterior capsule was measured.

### 2.6. Statistical Analysis

Statistical analyses were performed using IBM SPSS Statistics v27.0 (IBM Corp., Armonk, NY, USA). Because no previous data exist on intraoperative capsular-bag fluctuation using AFS versus GFS, a preliminary sample-size estimate was calculated assuming an anterior-chamber-depth percent change of 15% with AFS versus 30% with GFS after entering the phaco-probe with irrigation (M2), with an assumed SD of 25% for the paired percent-change differences. Since no prior in vivo data were available, this estimate was assumption-based and intended for planning this exploratory study. Under these assumptions, a total sample size of 28 paired subjects (56 eyes) would provide 80% power at a two-sided α = 0.05 in a paired design; with an anticipated 7-patient drop-out, 35 paired subjects (70 eyes) were deemed sufficient for this exploratory pilot trial. Given the feasibility focus, this calculation served only to illustrate expected variance. All intraoperative outcomes were analyzed and reported as absolute axial distances (mm), reflecting the direct measurement output of the iOCT scans.

Analyses were conducted on intra-individual pairs (AFS vs. GFS) using only complete paired data for a given measurement timepoint. The primary clinical endpoint was the anterior-capsule paired difference at M2 (phacotip insertion with irrigation on) between AFS and GFS within subjects, as gravity-based systems require higher preset irrigation pressure to compensate for anticipated vacuum demand, whereas adaptive fluidics allow for a lower initial intraocular pressure. Other timepoints and VAS scores were analyzed exploratorily and interpreted descriptively using the same paired approach. All outcomes derived from intraoperative SS-OCT were continuous measures (mm), while VAS scores were ordinal. Descriptive statistics included mean ± standard deviation (SD) and median (range). Normality of paired differences was verified using the Shapiro–Wilk test. Depending on distribution, either two-sided paired *t*-tests or Wilcoxon signed-rank tests were applied. Mean paired differences, 95% confidence intervals, and *p* values are reported. Additionally, Pearson correlation coefficients were calculated to determine the strength of inter-eye relationships at different timepoints. In case of non-normally distributed data, the Wilcoxon signed-rank test and the Spearman correlation coefficient were employed. A *p* value of less than 0.05 was considered statistically significant for the primary endpoint.

## 3. Results

Seventy eyes of 35 patients that met the inclusion criteria were enrolled in this randomized exploratory study Figure 1. One patient was excluded from the trial due to withdrawal of consent, four more patients had to be excluded as a different IOL implant was used, and three were excluded due to software errors with the prototype iOCT. The demographic baseline characteristics of the final 27 study participants are tabulated in Table 1.

**Figure 3:**
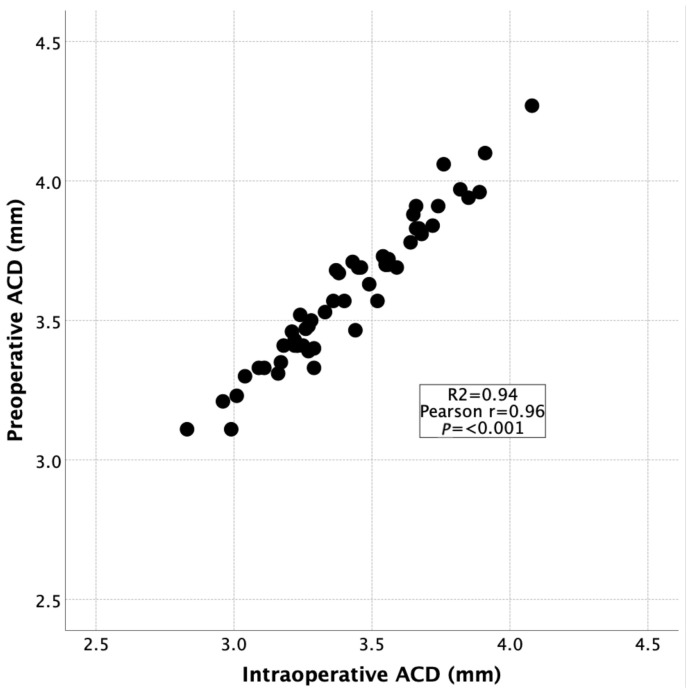
Correlation of preoperative SS-OCT-based ACD measurements and intraoperative SS-OCT-based ACD measurements. ACD = anterior chamber depth.

As expected, preoperative ACD measurements and intraoperative baseline ACD measurements exhibited a high correlation (r = 0.96, *p* < 0.001) as shown in Figure 3.

SS-OCT images during major phacoemulsification steps were successfully obtained. However, study-related measurements exhibited failure rates related to the intrinsic properties of iOCT imaging and the surgical setting (Table S1). For successful acquisitions, only cases with analyzable scans in both eyes were included in paired analyses, resulting in lower effective sample sizes for statistical comparison. The gross paired-eye measurement failure rate was thereby 39.5%, with higher failure rates observed during dynamic surgical phases, particularly during irrigation and instrument manipulation.

**Figure 4:**
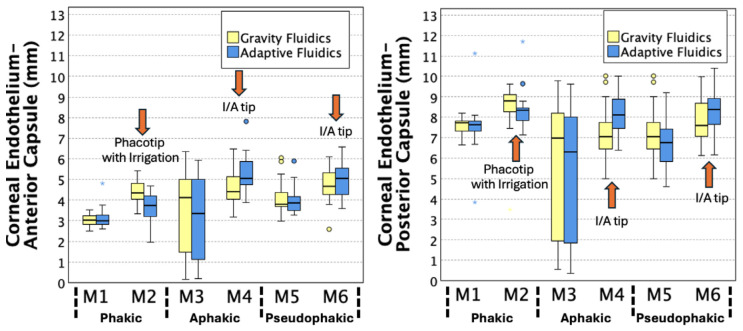
Boxplots showing intraoperative anterior (**left**) and posterior capsule (**right**) measurements. Gravity fluidics (yellow) and adaptive fluidics (blue) at measurement timepoints M1–M6.

Successful intra-individual intraoperative biometric measurements with GFS versus AFS with corresponding mean, median, and range values are shown in Table 2 and Figure 4. For both fluidics systems, measurements exhibited overall similar mean/medians with corresponding SDs throughout all timepoints. Aphakic anterior/posterior capsule OCT scans revealed by far the highest intra-individual variation. Measurements between both eyes of a subject were significantly correlated at the onset of surgery at the baseline timepoint M1 (r = 0.76, *p* ≤ 0.05, anterior capsule; r = 0.40, *p* ≤ 0.05, posterior capsule). During surgery, strong intra-individual correlations were observed at M3, in the aphakic state before entering with the I/A tip (r = 0.80, *p* ≤ 0.05, anterior capsule; r = 0.72, *p* ≤ 0.05, posterior capsule) and moderately at M5 in the pseudophakic state with OVD in place (r = 0.42, *p* ≤ 0.05, anterior capsule; r = 0.41, *p* ≤ 0.05, posterior capsule).

For axial anterior capsule distances, measurements after phacotip insertion (M2) revealed a numerical mean difference of 0.54 mm, with greater deepening under GFS than under AFS, which, however, was not statistically significant after Bonferroni adjustment (*P*_adj = 0.12). Otherwise, the remaining intra-individual mean anterior capsule distances were remarkably similar in terms of variability—although some outliers skewed the distribution of the data.

For posterior capsule distances, measurements also showed non-significant deepening with GFS against AFS at phaco-probe insertion (M2). Conversely, after phacoemulsification, distances between the corneal endothelium and posterior capsule did not reach statistical significance when using GFS versus AFS with I/A tip insertion.

Although the mean subjective discomfort scores were numerically lower in the AFS group after entering the phaco-probe with irrigation on, no statistically significant differences in patient discomfort were observed between the two fluidics systems at any timepoint (Table 3).

## 4. Discussion

Contemporary adaptive fluidics devices regulate IOP by employing a proactive infusion pressure increase using air pressure in the bottle according to the commanded vacuum level pressure. Although gravity fluidics and active fluidics have been compared extensively in the past in laboratory settings [5,25], in terms of cumulated dissipated energy, or in terms of postoperative visual recovery speed [7], no study has comparatively evaluated intraoperative biomorphometric dynamics during cataract surgery yet. Therefore, the aim of this exploratory study was to investigate capsular-bag dynamics in cataract patients via intraoperative SS-OCT using GFS versus AFS from the same manufacturer. Our rationale for selecting myopic eyes was that their deeper ACDs and tendency to have iris–lens diaphragm retropulsion syndrome with more pronounced intraoperative capsular bag oscillations lend themselves for such a comparative investigation. Also, myopic patients sometimes report significant discomfort when entering the eye with instruments using irrigation, such as the phaco-probe and the I/A tip.

Our study suggests that intraoperative OCT measurements are feasible for assessing capsular bag stability during cataract surgery, even though the surgical setting prohibited a complete set of intra-individual measurements in a substantial number of eyes. We attribute this not only to a learning effect when using the prototype, but also to OCT-related artifacts after irrigation obscuring posterior structures and poorly visible anterior capsular bag planes after rhexis and lens removal. Early failures were mainly related to suboptimal microscope–OCT alignment after instrument exchange, loss of signal from irrigation-related specular reflections, and motion artifacts during probe insertion.

Nevertheless, our study showed reduced capsular bag deepening when entering with AFS compared to GFS at the initiation of phacoemulsification (mean difference: 0.54 mm). This comparison was not statistically significant when adjusted for the multiple exploratory measurements across different timepoints (*p* = 0.12). Still, those changes relative to the baseline measurements (GFS mean difference to baseline: 1.19 mm; AFS mean difference to baseline: 0.63 mm) may indicate a potential advantage of AFS for anterior chamber stability when IOP differences are more pronounced. This is physiologically plausible, given that adaptive-fluidics systems allow for a lower starting IOP, whereas GFS necessitates a higher initial pressure to anticipate IOP drops that would occur with increasing outflow at subsequent surgical steps.

For other surgical timepoints, although mean distances and standard deviations between the corneal endothelium and the anterior/posterior capsule in the phakic, aphakic, and pseudophakic state were similar to our earlier trial via intraoperative partial coherence interferometry [24], intra-individual variability was remarkably similar between both study arms. Large variability was seen after exiting the eye after phacoemulsification, where the eye is not pressurized until again entered with the I/A tip shortly thereafter. The depth of the AC depends on the fluid egression when leaving the eye with the phaco-probe, which may vary greatly between eyes. Importantly, this highlights that phacoemulsification under AFS does not appear to sacrifice capsular bag stability for maintained physiological IOP compared to GFS. Those similarities in capsular-bag dynamics at more physiological pressures are relevant, because (1) IOP can usually not be directly measured in daily clinical practice, and (2) this would indicate that high IOP settings for the sake of capsular bag stability are not required.

We did, however, observe that anterior and posterior capsule distances tended to be, on average, moderately less deep under GFS in the aphakic and pseudophakic state (Table 2 and Figure 4). A possible explanation could be that myopic eyes with weakened zonules are known to exhibit a higher likelihood of low IOP during cataract surgery as recently evaluated by Wang et al. [26]. Axial myopia is prone to deep ACDs, floppier capsular bags, and weaker zonular fibers, and this could lead to increased infusion pressure resulting in BSS streaming into the liquefied vitreous cavity through the anterior hyaloid face. We hypothesize that in some cases, this fluid misdirection might have had a more pronounced effect on IOP lowering under GFS compared to the proactively regulated IOP under AFS.

Furthermore, although overall patient discomfort was slightly lower in the AFS group (VAS pain-score = 2.51 ± 1.73, GFS; VAS pain score = 2.33 ± 1.59, AFS; Table 3), no statistically significant differences were revealed in our trial. Therefore, our data do not demonstrate a measurable reduction in intraoperative discomfort with adaptive fluidics in this intra-individual pilot design. This contrasts with a recent randomized clinical study by Luo et al., which showcased a significantly improved active fluidics efficiency and subjective discomfort perception in 107 patients compared to GFS [27]. Similarly, another recent randomized trial by Liu et al. also found less pain perception with active fluidics, although study measurements were not conducted intra-individually [11]. Larger studies with sample sizes specifically calculated for subjectively experienced patient discomfort could offer additional insights whether the observed trend was truly non-significant. This would be interesting, since in our investigation the AFS group tended to have a lower mean VAS value at the initiation of phacoemulsification—where the difference in capsular bag deepening between both groups was most pronounced.

Several limitations should be considered when interpreting the present findings. First, while intraoperative baseline measurements with manual segmentation exhibited a high correlation with the nominal preoperative IOLMaster700 values (r = 0.96; Figure 3), the prototype did not incorporate image registration with invariant positional references adapted for other structures during the intraoperative setting. Indeed, participant attrition and the intraoperative image-acquisition failure rate reduced the number of complete paired observations available at several surgical stages which limited statistical power. Given the large intra/inter-individual variability, trends may be spurious (type I error) or missed in other stages due to limited power (type II error). Second, it must be emphasized that all three surgeons were highly experienced, and intraoperative hydrodynamics during phacoemulsification or irrigation/aspiration and related measurable differences between GFS and AFS might become more marked when adjusted for surgeons’ experience. Third, a successful acquisition of preoperative SS-OCT-based optical biometry was required for study inclusion, which may have resulted in underrepresentation of eyes with mature cataracts. Fourth, our study was not powered to detect a significant difference in VAS scores and was not designed to assess intraoperative or postoperative complication rates. Therefore, no direct conclusions can be drawn regarding comparative postoperative safety of AFS and GFS, as no systematic postoperative follow-up of corneal, iris, retinal, or visual outcomes was performed.

Despite these limitations, OCT-based visualization of phacodynamics may benefit from recently introduced intraoperative SS-OCT engines with considerably larger field of view, quasi-real-time visualization and volumetric parametrization, as well as potential assistive functions [22]. Notwithstanding that various previous studies have employed different phaco-machines when assessing active fluidics against GFS, a conventional efficiency metric such as the cumulative dissipated energy has either shown large energy conservation ranges for active fluidics or minimal differences [27,28,29,30]. Newer SS-OCT systems with surgical microscope integration will soon be commercialized and would lend themselves for future large-scale studies including differing patient demographics with larger sample sizes. Dynamic monitoring between the phacoemulsification tip and intraocular structures would be of particular interest in short eyes and patients at risk for corneal endothelial dysfunction. Since corneal endothelial cells are known to be sensitive to pressure-related damage, assessing capsular bag fluctuation via SS-OCT under different hydrodynamic conditions should therefore be endorsed to offer clinically relevant insights in those patient demographics in the future.

## 5. Conclusions

In conclusion, intraoperative SS-OCT enabled real-time visualization and partial quantitative assessment of capsular-bag dynamics during cataract surgery, demonstrating early feasibility of this imaging approach. The starting pressure under adaptive fluidics was associated with less anterior chamber deepening at the predefined primary endpoint during phaco-probe insertion; however, this comparison was not statistically significant after adjustment across the six measurement stages, and capsular-bag behavior remained comparable between both systems. Given the high measurement failure rate, the approach cannot yet be considered fully implementable for routine use, and these preliminary findings require confirmation in larger, adequately powered studies. Further technical refinement and workflow integration are required to achieve reliable continuous intraoperative imaging and support future randomized controlled trials comparing fluidics systems across broader patient populations.

## Figures and Tables

**Table 1:** Demographic data of study participants.

Parameter	Mean ± SD (Range)		*p*	r
Age	66 ± 10 (45–81)			
	GFS	AFS		
AL, mm	26.26 ± 0.96 (25.06–29.67)	26.29 ± 0.86 (25.07–29.30)	0.98 ^b^	0.53 †
PreOP ACD, mm	3.45 ± 0.25 (2.96–3.96)	3.45 ± 0.29 (2.83–4.08)	0.86 ^a^	0.71 *
PreOP LT, mm	4.56 ± 0.27 (3.89–4.96)	4.51 ± 0.26 (3.97–4.94)	0.46 ^a^	0.44 *
K, D	42.70 ± 1.75 (38.92–46.69)	42.59 ± 1.64 (38.69–46.24)	0.70 ^a^	0.49 *

ACD = anterior chamber depth; AFS = adaptive fluidics system; AL = axial length; D = diopter; GFS = gravity fluidics system; LT = lens thickness; K = keratometry; PreOP = preoperative; SD = standard deviation; ^a^ paired two-tailed *t*-test; ^b^ two-tailed Wilcoxon signed rank test; * Pearson correlation significant at *p* < 0.05, † Spearman correlation significant at *p* < 0.05.

**Table 2:** Intraoperative SS-OCT measurements during phacoemulsification.

Parameter (mm)	Mean ± SD; Median (Range)		*P*	*P*_adj	r	n
	GFS	AFS				
Anterior capsule						
M1 (Baseline)	3.04 ± 0.27; 3.03 (2.49–3.52)	3.09 ± 0.44; 3.01 (2.60–4.81)	0.41 ^b^	1.00	0.76 †	27
M2 (Phakic + Irrigation)	4.23 ± 0.58; 4.35 (3.33–5.43)	3.72 ± 0.73; 3.74 (2.47–4.70)	0.02 ^a^	0.12	0.12	18
M3 (Aphakic)	3.46 ± 2.06; 4.13 (0.16–6.36)	3.30 ± 2.12; 3.34 (0.20–5.95)	0.65 ^a^	1.00	0.80 †	15
M4 (Aphakic + I/A tip)	4.62 ± 0.92; 4.40 (3.17–6.47)	5.32 ± 1.05; 5.05 (3.87–7.81)	0.16 ^a^	0.96	−0.08	10
M5 (Pseudophakic)	4.06 ± 0.79; 3.80 (2.98–6.05)	3.92 ± 0.49; 3.87(3.26–5.89)	0.38 ^b^	1.00	0.42 †	22
M6 (Pseudophakic + I/A tip)	4.63 ± 0.83; 4.68 (2.59–6.09)	4.99 ± 0.81; 5.07 (3.60–6.59)	0.14 ^a^	0.84	0.22	18
Posterior capsule						
M1 (Baseline)	7.59 ± 0.38; 7.72 (6.66–8.19)	7.54 ± 1.07; 7.63 (3.84–11.12)	0.80 ^b^	1.00	0.40 †	27
M2 (Phakic + Irrigation)	8.30 ± 1.35; 8.60 (3.48–9.62)	8.36 ± 1.05; 8.14 (7.12–11.70)	0.12 ^a^	0.72	0.46	17
M3 (Aphakic)	5.54 ± 3.42; 6.98 (0.56–9.78)	5.40 ± 3.37; 6.31(0.36–9.64)	0.79 ^a^	1.00	0.72 †	17
M4 (Aphakic + I/A tip)	7.60 ± 1.36; 7.14 (5.52–10.80)	8.38 ± 1.47; 8.12 (6.37–11.91)	0.22 ^a^	1.00	−0.19	13
M5 (Pseudophakic)	7.21 ± 1.25; 6.98 (4.99–10.02)	6.77 ± 0.95; 6.75 (4.60–9.22)	0.12 ^a^	0.72	0.41 †	21
M6 (Pseudophakic + I/A tip)	7.79 ± 1.08; 7.55 (6.13–9.98)	8.34 ± 1.04; 8.48 (6.17–10.40)	0.07 ^b^	0.42	0.37	18

AFS = adaptive fluidics system; GFS = gravity fluidics system; SD = standard deviation; ^a^ paired two-tailed *t*-test; ^b^ two-tailed Wilcoxon signed rank test; † correlation coefficient significant at <0.05. *P*_adj = Bonferroni-adjusted *p* values across the six measurement stages.

**Table 3:** Subjective patient discomfort.

Parameter (VAS)	Mean ± SD; Median (Range)		*p* *
	GFS	AFS	
Phakic + Irrigation	2.06 ± 1.71; 1 (0–7)	1.51 ± 1.72; 1 (0–6)	0.12
Aphakic + I/A tip	2.66 ± 1.89; 3 (0–7)	2.39 ± 1.98; 2 (0–7)	0.52
Pseudophakic + I/A tip	3.03 ± 1.98; 3 (0–8)	2.63 ± 1.85; 2 (0–8)	0.36
Overall	2.51 ± 1.73; 1 (0–6)	2.33 ± 1.59; 2 (0–6)	0.70

AFS = adaptive fluidics system; GFS = gravity fluidics system; SD = standard deviation; VAS = Visual analog scale, * two-tailed Wilcoxon signed rank test significant at *p* < 0.05.

## Data Availability

The original contributions presented in this study are included in the article/Supplementary Materials. Further inquiries can be directed to the corresponding author.

## Supplementary Materials

The following supporting information can be downloaded at: https://www.mdpi.com/article/10.3390/diagnostics16142282/s1, Table S1: Intraoperative SS-OCT measurement failure rate during phacoemulsification.

## Abbreviations

The following abbreviations are used in this manuscript:

ACD	anterior chamber depth
AFS	adaptive fluidics system
AL	axial length
BSS	balanced salt solution
D	diopter
GFS	gravity-based fluidics system
I/A	irrigation/aspiration
IOL	intraocular lens
IOP	intraocular pressure
iOCT	intraoperative optical coherence tomography
K	keratometry
LT	lens thickness
OCT	optical coherence tomography
OVD	ophthalmic viscosurgical device
SD	standard deviation
SS-OCT	swept-source optical coherence tomography
VAS	visual analog scale
