# Supplementary material for: Feasibility of Intraoperative SS-OCT for Assessing Capsular-Bag Dynamics During Cataract Surgery: Pilot Randomized Intra-Individual Comparison of Adaptive Versus Gravity-Based Fluidics
Source: Diagnostics (Basel). 2026 Jul 21;16(14):2282. doi: 10.3390/diagnostics16142282 (PMC13407691; doi:10.3390/diagnostics16142282)
Supplement: Supplementary file 1 [file diagnostics-16-02282-s001.zip › diagnostics-4430124-supplementary.pdf]

Table S1. Intraoperative SS-OCT measurement failure rate during phacoemulsification						
Timepoint	Total	Successful	Failed	Failure rate (%)	Paired anterior	Paired posterior
M1 (Baseline)	54	54	0	0	27	27
M2 (Phakic+Irrigation)	54	36	18	33	18	17
M3 (Aphakic)	54	34	20	37	15	17
M4 (Aphakic+I/A tip)	54	30	24	44	10	13
M5 (Pseudophakic)	54	46	8	15	22	21
M6 (Pseudophakic+I/A tip)	54	36	18	33	18	18
